# Supplementary material for: Two-photon imaging of neuronal activity in motor cortex of marmosets during upper-limb movement tasks
Source: Nat Commun. 2018 May 14;9:1879. doi: 10.1038/s41467-018-04286-6 (PMC5951821; doi:10.1038/s41467-018-04286-6)
Supplement: Supplementary file 1 — Supplementary Information [file 41467_2018_4286_MOESM1_ESM.pdf]

Supplementary Information

Two-photon imaging of neuronal activity in motor cortex of marmosets during upper-limb movement tasks

Teppei Ebina, Yoshito Masamizu, Yasuhiro R. Tanaka, Akiya Watakabe, Reiko Hirakawa, Yuka Hirayama, Riichiro Hira, Shin-Ichiro Terada, Daisuke Koketsu, Kazuo Hikosaka, Hiroaki Mizukami, Atsushi Nambu, Erika Sasaki, Tetsuo Yamamori, and Masanori Matsuzaki

SUPPLEMENTARY FIGURES

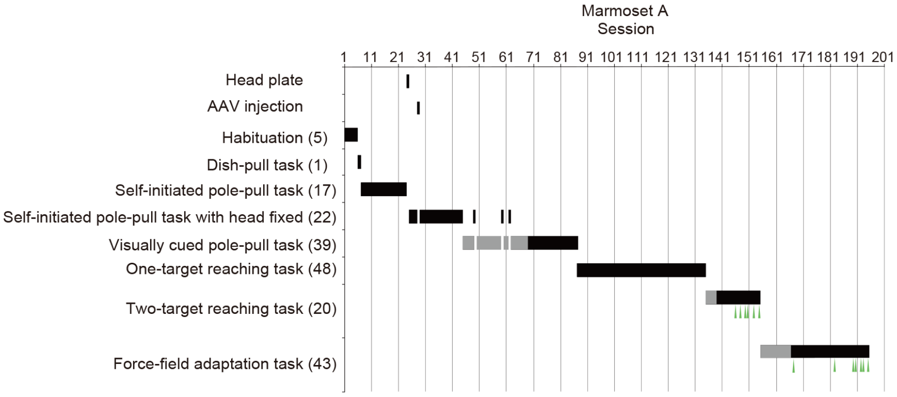

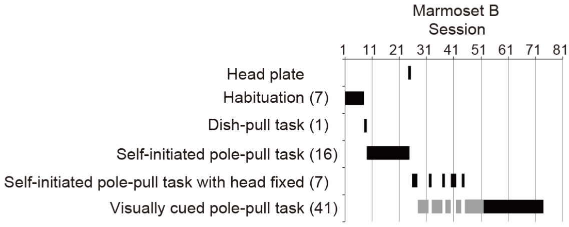

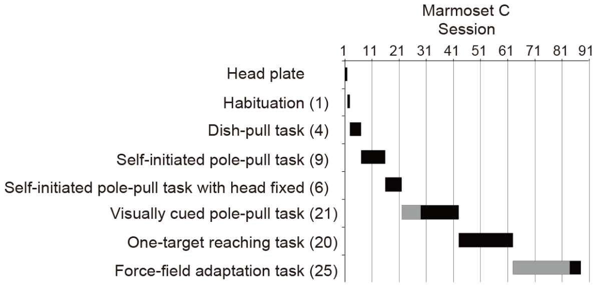

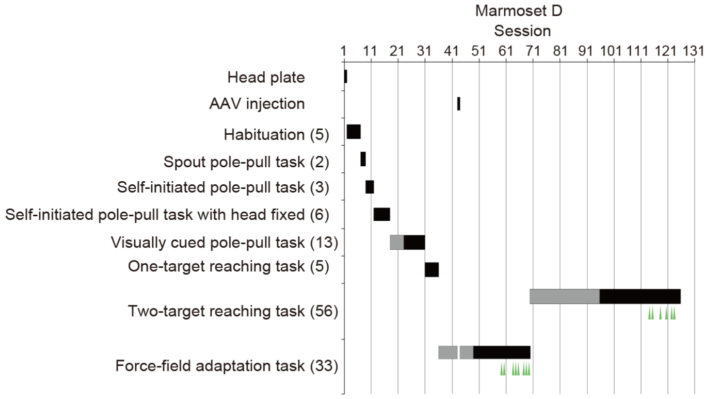

Supplementary Figure 1. The task schedules for the four marmosets.

Black and gray bars indicate the training sessions for the tasks, as indicated on the left. Gray bars indicate the durations over which the task parameters were changed. The numbers in parentheses on the left indicate the total session number for the corresponding task. The total number of training sessions performed without head fixation ranged from 10 to 24. The total number of training sessions of the self-initiated pole-pull task with head fixation ranged from 6 to 22. Green arrowheads represent the sessions with imaging experiments.

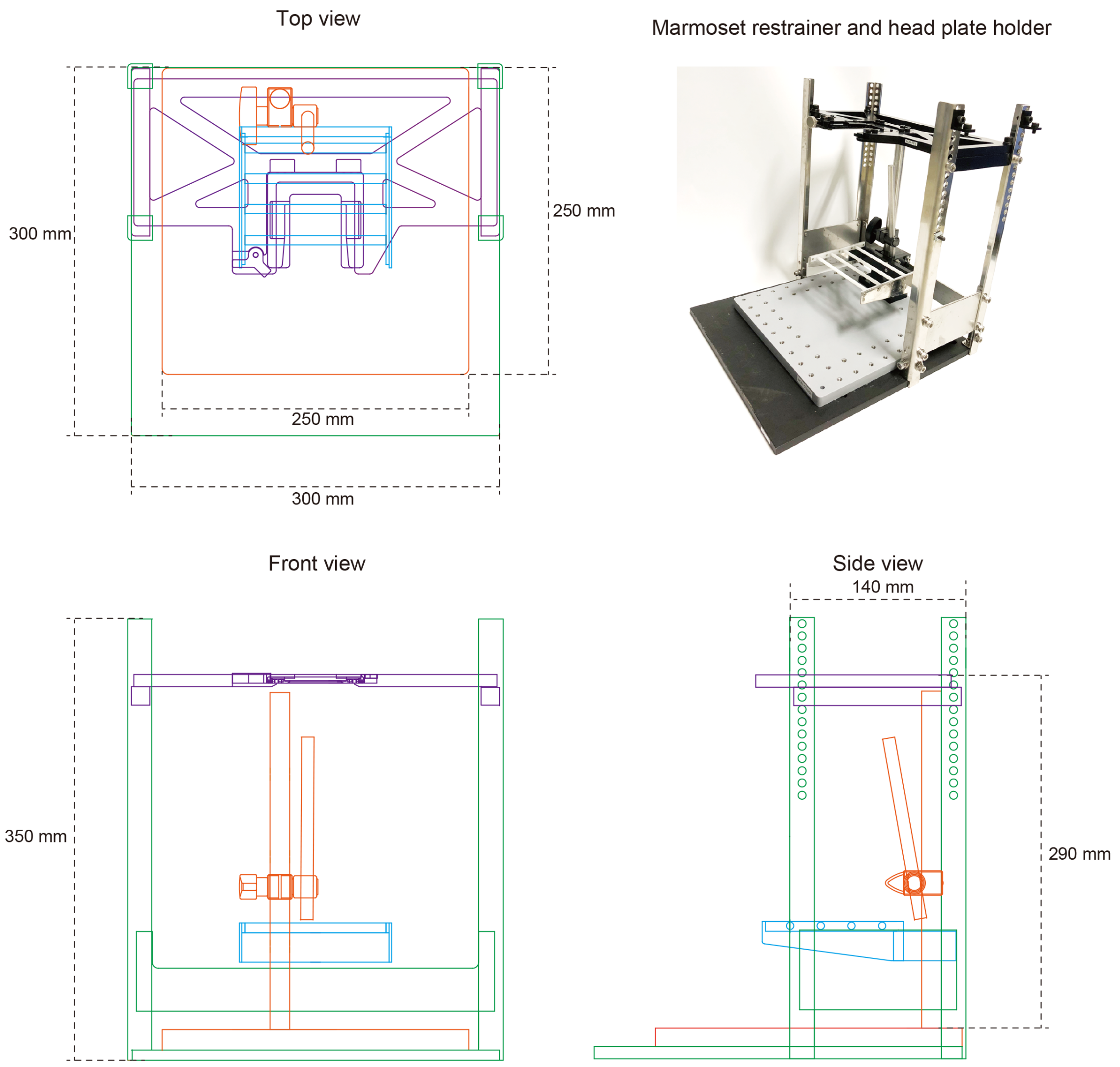

Supplementary Figure 2. Projection views of the marmoset restrainer and head plate holder. Identical colors indicate the same part: orange, restrainer; light blue, foot rest; purple, head plate holder; green, frame of head plate holder.

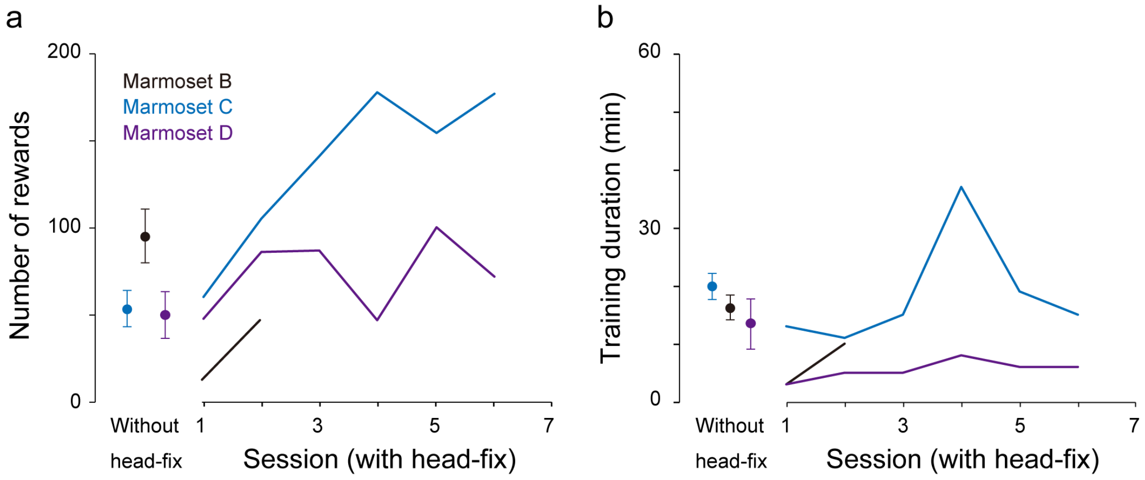

Supplementary Figure 3. Training performances in the self-initiated pole-pull task with and without head fixation.

(a) The number of reward deliveries in the pole-pull task without and with head fixation in three marmosets. The session-averaged values in the task without head fixation are shown as mean ± SEM (n = 16, 9, and 3 sessions for marmosets B, C, and D, respectively). The values in the task with head fixation are plotted against the training sessions before other tasks started. Marmoset A was not included in this figure, as the reward number was not recorded until session 12 of the head-fixed pole-pull task. (b) The training duration in the pole-pull task without and with head fixation. Marmoset D took less time to acquire a similar number of rewards when its head was fixed than when it was not fixed, as it appeared to concentrate harder on the task when its head was fixed.

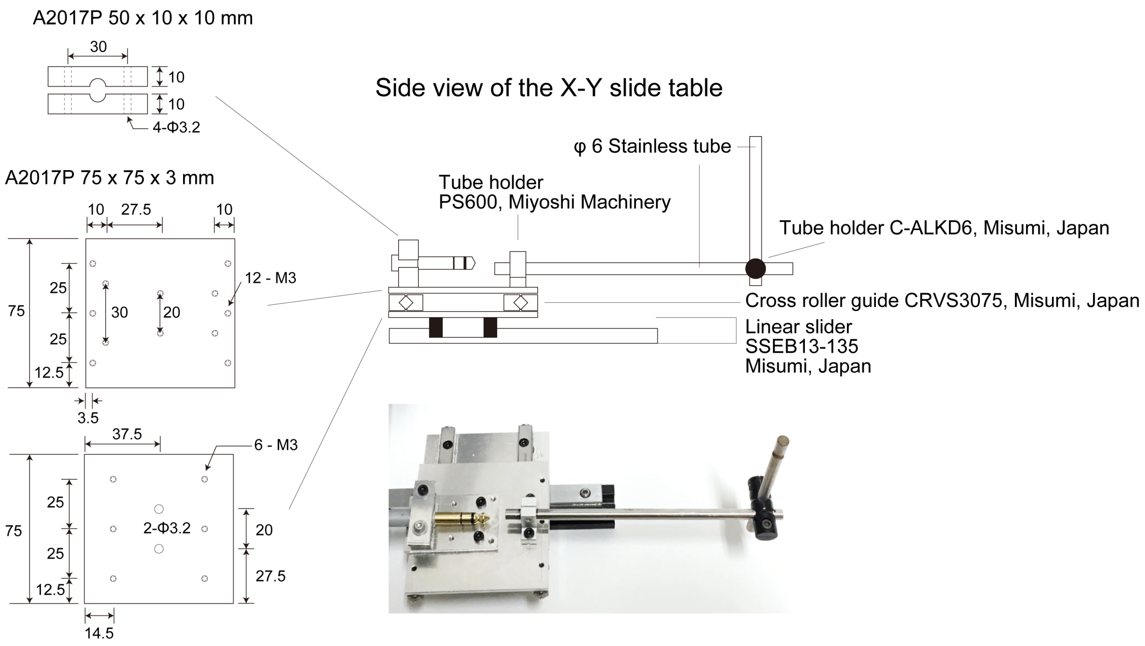

Supplementary Figure 4. Projection views of the X-Y slide table with the pole connected to the robotic arm.

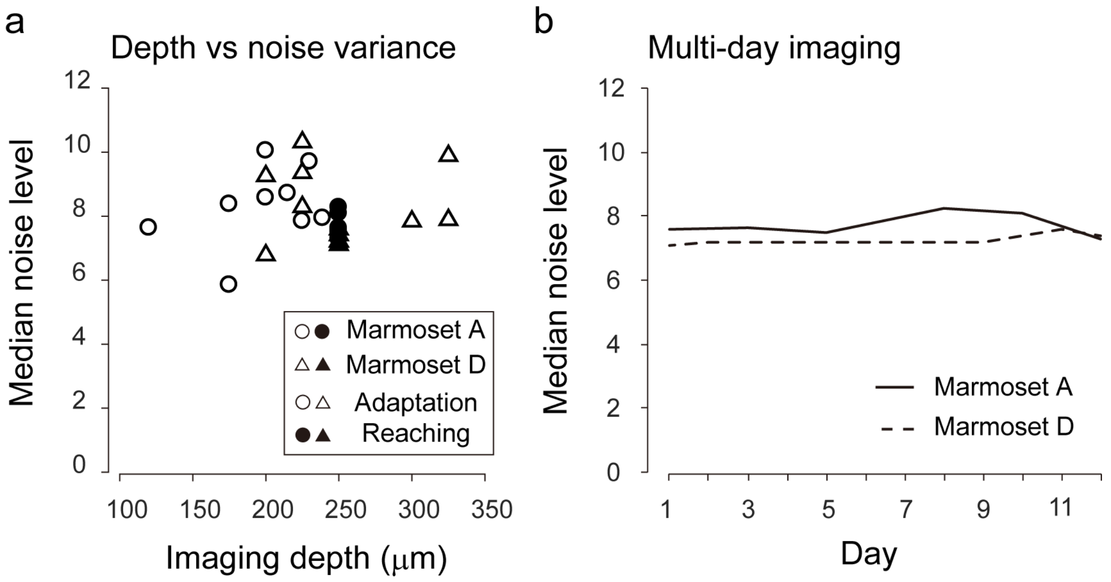

Supplementary Figure 5. Noise level of the imaging in each field.

(a) Relationships between the imaging depth and the noise level. The noise level was estimated as the median of the standard deviation in the high-frequency components of the motion-corrected fluorescence signals at each pixel, as calculated by the constrained_foopsi script in the CNMF package. Open circles and open triangles represent the imaging fields during the force-field adaptation task in marmosets A and D, respectively. Filled circles and filled triangles represent the imaging fields during the two-target reaching task in marmosets A and D, respectively. (b) The noise level during the two-target reaching task was plotted against the imaging day. Lines and dotted lines indicate fields from marmosets A and D, respectively. Correlation coefficients between the number of days and the median noise level were −0.09 (P = 0.91) for marmoset A and 0.77 (P = 0.10) for marmoset D.

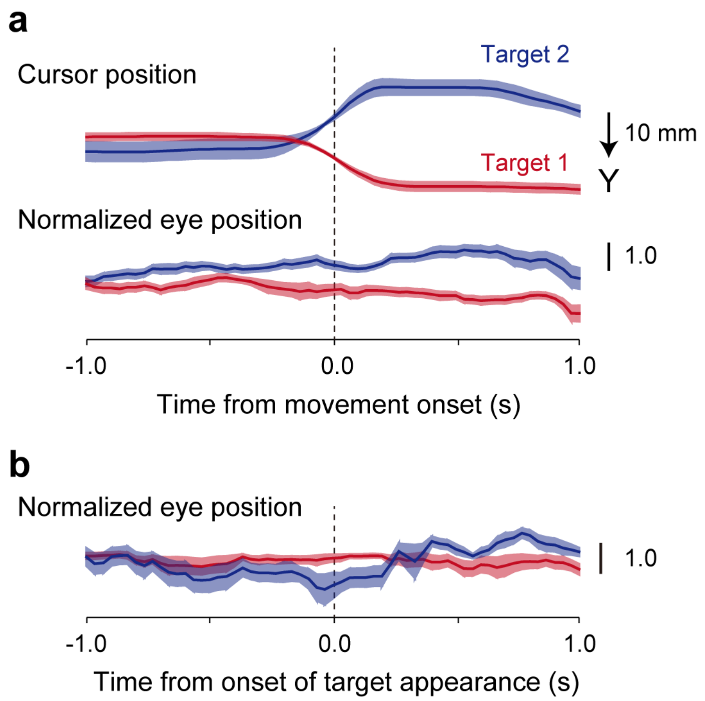

Supplementary Figure 6. Averaged eye movements during simultaneous two-photon imaging and two-target reaching task performance.

(a) Representative trial-averaged trajectories of Y-axis cursor position (top) and normalized Y-axis eye position (bottom) during simultaneous two-photon imaging and two-target reaching task performance in session 18 (day 10). The trajectories were aligned to the time at which the cursor traversed the fixation square towards target 1 (red) or 2 (blue). Normalization was performed by calculating the Z-score of the eye position within the period of the imaging series. Lines and shaded areas represent mean ± SEM (target 1, n = 12 successful trials; target 2, n = 8 successful trials). (b) Representative trial-averaged trajectories of the normalized Y-axis eye position aligned to the time at which either target appeared on the screen. Other conventions are the same as in a.

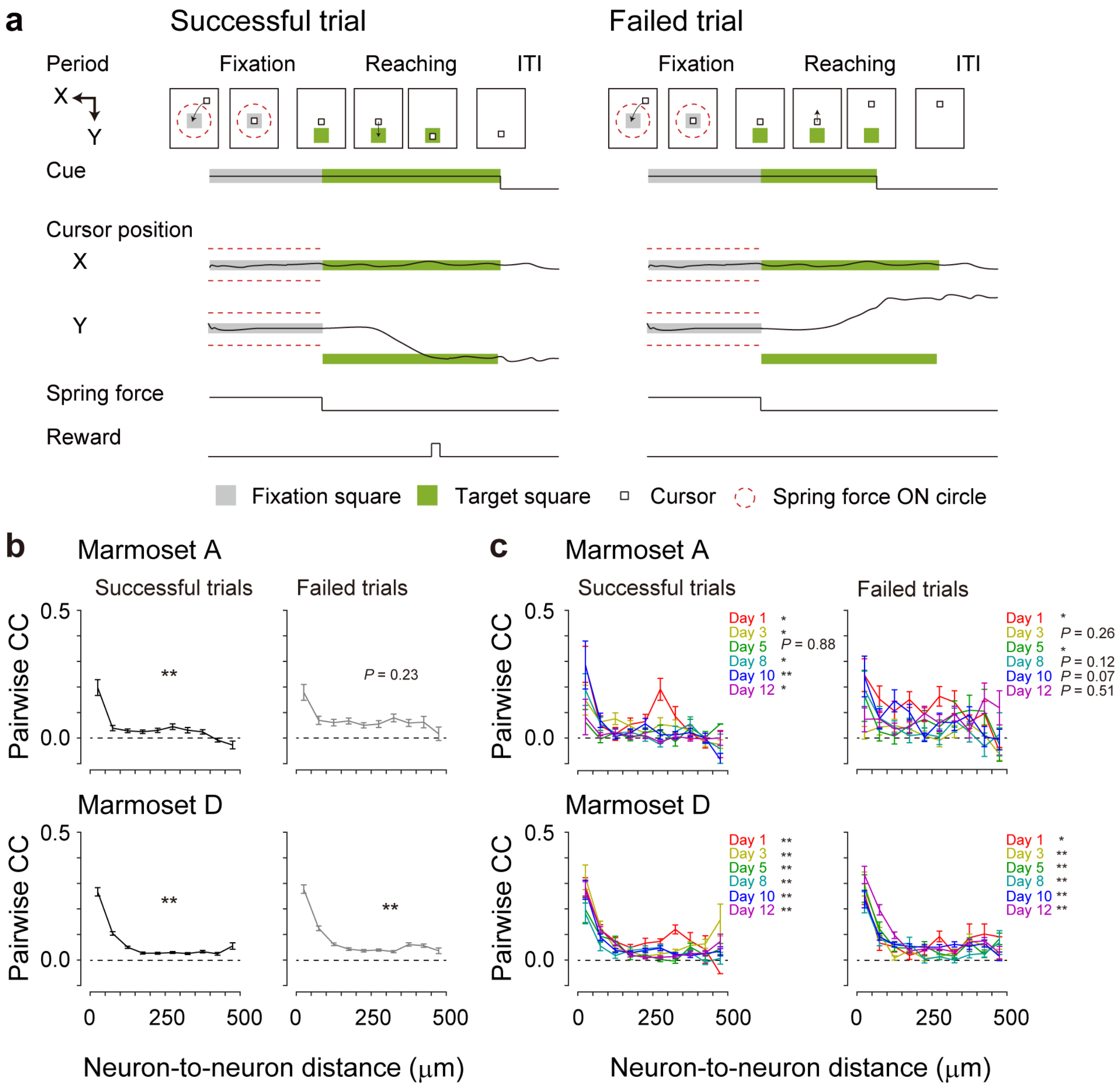

Supplementary Figure 7. Relationships between the pairwise correlation coefficients in Δ*F*/*F* signals and cellular distance.

(a) A trial was composed of fixation and reaching periods and an ITI. Left, a trial when reaching an appropriate target was rewarded (successful trial). Right, a trial when reaching was not appropriate and a reward was not given (failed trial). (b) The relationships between pairwise correlations in the activity during the successful (left) or failed (right) trial periods and the distance between pairs of neurons. Top, marmoset A. Bottom, marmoset D. Pearson correlation coefficients were calculated for each pair of denoised Δ*F*/*F* of neurons as the pairwise correlation coefficients (pairwise CCs).

The pairwise CCs were pooled across imaging days. Spearman CCs between the pairwise CCs and distances were statistically significant in both successful and failed trials (*$P < 0.05$ and **$P < 0.01$). (c) The relationships in the successful (middle) and failed trials (right) on each imaging day. Different colors indicate different imaging days. The *P* values for the Spearman correlation coefficients are shown in the top-right of each panel (*$P < 0.05$ and **$P < 0.01$). The conventions are the same as in b.

LEGENDS FOR SUPPLEMENTARY MOVIES

Supplementary Movie 1. Representative behavior of a head-fixed marmoset in session 1 of a one-target reaching task.

Marmoset A had to control the manipulandum to move the cursor to the green target and hold it for 100 ms to obtain a reward, but failed. The movie is a 30 s real-speed movie.

Supplementary Movie 2. Representative behavior of a head-fixed marmoset in session 9 of a one-target reaching task.

Marmoset A successfully controlled the manipulandum to move the cursor to the green target in a straight trajectory and hold it there for 200 ms to obtain a reward. The movie is a 30 s real-speed movie.

Supplementary Movie 3. Two-photon calcium imaging of multiple cortical neurons at day 1 during a two-target reaching task.

Motion-corrected movie at 10× speed. Each image is the average of 60 frames. The imaging field is the same as the top image in Fig. 7a. The white square in the right panel represents the cursor, and green and gray boxes indicate the positions of the target and fixation squares, respectively. Target 1 (upper target) or target 2 (bottom target) was randomly displayed in each trial.

Supplementary Movie 4. Two-photon calcium imaging of multiple cortical neurons at day 10 during a two-target reaching task.

Motion-corrected movie at 10× speed. Each image is the average of 60 frames. The imaging field is the same as in the bottom image in Fig. 7a. Other conventions are the same as in Supplementary Movie 3.
