# Supplementary material for: Two-photon imaging of neuronal activity in motor cortex of marmosets during upper-limb movement tasks
Source: Nat Commun. 2018 May 14;9:1879. doi: 10.1038/s41467-018-04286-6 (PMC5951821; doi:10.1038/s41467-018-04286-6)
Supplement: Supplementary file 3 — Description of Additional Supplementary Files [file 41467_2018_4286_MOESM3_ESM.pdf]

Description of Additional Supplementary Files

File Name: Supplementary Movie 1
Description: Marmoset A had to control the manipulandum to move the cursor to the green target and hold it for 100 ms to obtain a reward, but failed. The movie is a 30 s real-speed movie.

File Name: Supplementary Movie 2
Description: Marmoset A successfully controlled the manipulandum to move the cursor to the green target in a straight trajectory and hold it there for 200 ms to obtain a reward. The movie is a 30 s real-speed movie.

File Name: Supplementary Movie 3
Description: Motion-corrected movie at 10× speed. Each image is the average of 60 frames. The imaging field is the same as the top image in Fig. 7a. The white square in the right panel represents the cursor, and green and gray boxes indicate the positions of the target and fixation squares, respectively. Target 1 (upper target) or target 2 (bottom target) was randomly displayed in each trial.

File Name: Supplementary Movie 4
Description: Motion-corrected movie at 10× speed. Each image is the average of 60 frames. The imaging field is the same as in the bottom image in Fig. 7a. Other conventions are the same as in Supplementary Movie 3.
